# Fraxetin attenuates disrupted behavioral and central neurochemical activity in a model of chronic unpredictable stress

**DOI:** 10.3389/fphar.2023.1135497

**Published:** 2023-03-23

**Authors:** Zainab Ahmed, Ahmed Tokhi, Mehreen Arif, Naeem Ur Rehman, Vahid Sheibani, Khalid Rauf, Robert D. E. Sewell

**Affiliations:** ^1^ Department of Pharmacy, COMSATS University Islamabad, Abbottabad campus, Abbottabad, Pakistan; ^2^ Faculty of Pharmacy, Gomal University, Dera Ismail Khan, KP, Pakistan; ^3^ Neuroscience Research Center, Institute of Neuropharmacology, Kerman University of MedicalSciences, Kerman, Iran; ^4^ Cardiff School of Pharmacy and Pharmaceutical Sciences, Cardiff University, Cardiff, United Kingdom

**Keywords:** fraxetin, chronic unpredictable stress, ELISA, corticosterone, HPLC

## Abstract

**Purpose:** Chronic unpredictable stress (CUS) induces long-term neuronal and synaptic plasticity with a neurohormonal disbalance leading to the development of co-existing anxiety, depression, and cognitive decline. The side effects and delayed onset of current clinically used antidepressants has prompted a quest for antidepressants with minimum drawbacks. Fraxetin is a natural coumarin derivative with documented antioxidant and neuroprotective activity though its effects on stress are unknown. This study therefore aimed to investigate any possible acute effect of fraxetin in behavioral tests including a CUS paradigm in correlation with brain regional neurochemical changes.

**Methods:** Mice were subjected to a series of mild stressors for 14 days to induce CUS. Furthermore, behavioral performance in the open field test, forced swim test (FST), Y-maze and elevated plus-maze were evaluated. *Postmortem* frontal cortical, hippocampal and striatal tissues were analyzed *via* high-performance liquid chromatography (HPLC) for neurochemical changes.

**Result:** Acute administration of fraxetin (20–60 mg/kg, orally) decreased depression-like behavior in the FST and behavioral anxiety in both the open field test and elevated plus-maze. Memory deficits induced during the CUS paradigm were markedly improved as reflected by enhanced Y maze performance. Concurrent biochemical and neurochemical analyses revealed that only the two higher fraxetin doses decreased elevated serum corticosterone levels while diminished serotonin levels in the frontal cortex, striatum and hippocampus were reversed, though noradrenaline was only raised in the striatum. Concomitantly, dopamine levels were restored by fraxetin at the highest dose exclusively in the frontal cortex.

**Conclusion:** Acute treatment with fraxetin attenuated CUS-induced behavioral deficits, ameliorated the increased corticosterone level and restored altered regional neurotransmitter levels and this may indicate a potential application of fraxetin in the management of anxiety and depression modeled by CUS. However, further studies are warranted regarding the chronic effects of fraxetin behaviorally and neurochemically.

## 1 Introduction

Stress is generally a well-recognized and studied global problem of modern society (Carrasco and Van De Kar, 2003). It can be categorized as any state/threat, whether actual or perceived which may alter body homeostasis (Charmandari et al., 2005), and thus contribute to etiology and repetitive episodes of depression (Lee et al., 2002). It is well documented in clinical and preclinical studies that neuronal atrophy and decreased neurotransmitter levels may arise due to chronic stress in specific brain areas like the prefrontal cortex (PFC) and hippocampus, leading to both cognitive, affective and social decline (Rajkowska et al., 1999; Drevets et al., 2008; Sacher et al., 2012). Maintaining homeostasis in an aversive situation (involving stressors) requires the activation of a complex range of neurochemical, neuroendocrine and genetic factors coupled with perception and this is collectively termed the stress response. This results in several physiological and behavioral changes tending to increase survival chances in unfavorable conditions (Charmandari et al., 2005). Increased awareness, improved cognition and increased analgesia are the behavioral outcomes of acute stress, while increased cardiovascular tone, respiratory rate, decreased food intake, growth, immunity, and reproduction represent the physiological effects. Stress has dual consequences which may either be positive or negative in nature (Sapolsky et al., 2000; Habib et al., 2001). Acute stress usually occurs for a brief period and may protect the individual from any aversive condition by preparing the body for a “fight or flight” situation. In contrast, persistent and long-term stress may produce deleterious effects (Gold, 2015), *via* elevated cortisol levels that may result in dysregulated emotional, biological, as well as psychological ailments including anxiety, depression, stress-induced asthma, cardiomyopathy, irritable bowel syndrome, chronic headaches, and substance abuse (Stetler and Miller, 2011).

Stress is a normal body response to external or internal stressors identified by stress mediators implicating the endocrine, nervous and immune systems. Glucocorticoids and epinephrine play important roles in body homeostasis i.e., by adapting to the stressor response (Duman and Aghajanian, 2012). However, if this response persists for an extended episode, it not only disrupts normal homeostasis, but also undermines the body’s ability to cope with the stressor ultimately leading to chronic-stress illnesses like anxiety, depression, and cognitive deficits (Nestler et al., 2002; Bondi et al., 2008). In this context, unpredictable stressful events may even predispose individuals to the development of neuropsychiatric disorders (Nestler et al., 2002; Moghaddam and Javitt, 2012).

The clinically used antidepressants increase the monoamine levels in the synaptic cleft, and they include, selective serotonin reuptake inhibitors (SSRIs), serotonin and noradrenaline reuptake inhibitors (SNRIs), monoamine oxidase inhibitors (MAOIs), and tricyclic antidepressants (TCAs). However, they have a slow onset of action, poor tolerability and severe adverse effects including insomnia, dependency, a withdrawal syndrome, weight gain, migraine and suicidal tendencies (Blackburn, 2019). These limitations necessitate the development of newer antidepressants with improved efficacy, fast onset of action, safety, tolerability, reduced or no withdrawal syndrome and a contribution to overall mental health (Blackburn, 2019).

In this respect, plant-based natural products may provide an alternative solution and one such phytochemical class are the coumarins with recognized therapeutic potentials in multiple neuropsychiatric illnesses through antioxidant, neuroprotective, neuromodulator and antidepressant actions(Skalicka-Woźniak et al., 2016; Abu-Aisheh et al., 2019; Pruccoli, 2019; Daliev et al., 2021; Koyiparambath et al., 2021; Sharifi-Rad et al., 2021; Kılıç, 2022). One particular coumarin derivative, fraxetin, is an *O*-methylated coumarin (An et al., 2020), present in many functional foods as well as several dietary supplements with a documented neuroprotective profile mediated primarily through inhibition of oxidative stress (Pruccoli, 2019; Qin et al., 2019; Zhang et al., 2021a). Although the antioxidant, anti-inflammatory, and neuroprotective activities of fraxetin have been described (Zhang et al., 2021a), its effects on stress have not been reported in the literature and remain unknown. Consequently, the present study was undertaken to investigate the effect of fraxetin (20, 40, and 60 mg/kg) in the CUS model which is a widely used animal model to investigate depressive-like behavior. The behavioral activity in the current study was then correlated with brain regional neurochemical parameters in mice.

## 2 Materials and methods

### 2.1 Animals

Adult male BALB/c mice (n = 6/group) ranging in weight from 22 to 26 g) were used during experimentation. All animals were kept at room temperature (22 ± 2°C) on a 12/12 h of light/dark cycle and access to food and water was available *ad libitum*. The experimental protocol and animal welfare were approved by the Ethical Committee COMSATS University Islamabad, Abbottabad campus (ethical approval number PHM.Eth/CS-M01-017-1017) with guidelines adherence to UK Animals (Scientific Procedures) Act 1986.

### 2.2 Chemicals

Fraxetin (CAS: 574-84-5) was purchased from Shandong Chuangying Chemical Co., Ltd. China. Fluoxetine (A.R. No: FP/FX/1905002) was gifted by Palam Pharma PVT. Ltd.

### 2.3 Experimental design

Fraxetin (20, 40 and 60 mg/kg) (Li et al., 2011; Murali et al., 2013) as well as fluoxetine (10 mg/kg), Kryst et al. (2022) were administered orally (p.o.) in a single dose 5 h after day 14 of the CUS protocol. All the drugs were freshly prepared prior to administration. Animals were randomly allocated to the following six treatment groups (n = 6/group): 1) Non-stressed + normal saline, 2) stressed + normal saline (CUS model group) 3) stressed + fluoxetine (10 mg/kg), 4) stressed + fraxetin (20 mg/kg), 5) stressed + fraxetin (40 mg/kg) and 6) stressed + fraxetin (60 mg/kg).

### 2.4 Chronic unpredictable stress procedure

The CUS protocol was composed of different stressors applied daily at a variable times of the day over a 14-day period and it was designed to avoid the predictability of each stressor (Table 1). There were two groups of animals; CUS (stressed) and control (non-stressed) mice. During the 14 days, non-stressed animals were kept in their home cage according to the standard protocol while the stressed animals underwent 14 days of the variable stress paradigm. On day 14, all the stressed groups received fraxetin, fluoxetine, or vehicle except for the CUS only model group and subsequently, behavioral parameters were assessed (Moretti et al., 2012). Separate groups were subjected to behavioral testing, one hour after acute administration of fluoxetine, fraxetin or vehicle. In addition, the dosing and testing procedure was executed 5 h after of the last stressor, in order to avoid any additional stress experienced during behavioral testing which occurred.

**TABLE 1:** 14 days schedule of stressors in the chronic unpredictable stress model.

Days	Stressor	Duration
1	Restrain	1.5 h
2	Cold Swim (15°C)	10 min
3	Wet sawdust bedding/box housing tilted (45°)	16 h
4	Inversion of light/dark cycle	16 h
5	Tail pinch	10 min
6	Swim (25°C)	6 min
7	Restrain	1.5 h
8	Food and water deprivation	16 h
9	Cold swim (15°C)	10 min
10	Wet sawdust bedding/box housing tilted (45°)	24 h
11	Inescapable shock (0.7 mA, 0.5 s/min)	3 min
12	Inversion of light/dark cycle	16 min
13	Tail pinch	10 min
14	Inescapable shock (0.7 mA, 0.3 s/min)	3 min

### 2.5 Behavioral assessment

All behavioral tests were evaluated manually from video recordings by an experimenter who was blind to the treatments.

#### 2.5.1 Locomotor activity boxes (open field test)

The open-field test is a simple method used to assess underlying exploratory behavior in rodents. Each animal displayed simple movement or ambulation in an open field or more complex behaviors like rearing or thigmotaxis. Locomotor activity was assessed by placing the mice individually in an arena comprised of wooden monitor boxes divided internally by lines on the floor into equivalent quadrants 22.8 cm^2^ each and surrounded externally by a 45.6 cm high wall. The locomotor activity of the test animals was measured for 30 min. A digital CAT-I camera installed 230 cm above the locomotor arena was used for locomotion recordings and locomotor activity (simple ambulation) was assessed in activity monitoring boxes (45.6 × 45.6 cm × 30 cm), internally divided into four quadrants measuring 22.8 cm^2^ with floor line markings. After cleaning the arena with 70% alcohol, animals were introduced to the center of box, and the activity was recorded by a video camera mounted 230 cm above the box. The number of lines crossed in 30 min was noted (Moretti et al., 2011; Arif et al., 2022).

#### 2.5.2 Elevated plus maze (EPM)

This method is used to investigate anxiogenic and anxiolytic drug effects in laboratory animals and it is based on the survival instinct that rodents are generally aversed to open spaces because of increased exposure risk to predation.

The apparatus consisted of a “plus-shaped” maze elevated above the floor with alternating open and enclosed arms and an open central area. The method relies on the fact that rodents are generally aversed to exploration in open spaces. Single animals were gently placed in the central area, allowed to roam freely for 5 min and the time spent in open and enclosed arms was recorded (Handley and Mithani, 1984).

#### 2.5.3 Y-maze test (Y-maze)

The Y-maze is a widely used technique to study learning and short-term memory in rodents and it incorporates spontaneous alternation as a measure of spatial working memory.

The apparatus consisted of three equal-length arms positioned at an angle of 120° (21 cm long × 8.5 cm wide × 40 cm height). Each animal was positioned at the midpoint of the apparatus and permitted to freely explore all arms for 5 min. The time spent in each arm was recorded using a video camera and between each animal procedure, the apparatus was swabbed with 70% ethanol. Brain areas including the prefrontal cortex, hippocampus and basal forebrain are considered to be involved in this type of memory process. Following animal placement in the maze and consecutive arm entry, the percentage alternation between arms was calculated as follows:

$$\%\;alternation=\left(total\;number\;of\;alternations\right)\;/\;\left(no\;of\;entry\;in\;each\;arm\right)\;x\;100$$ (1)

Distance, speed, and time spent in every arm was also recorded using a CAT-I video camera (Golub et al., 2015).

#### 2.5.4 Forced swimming test (FST)

This test was originally developed by Porsolt et al. (1977) to screen compounds for prospective antidepressant activity in rodents. Mice were introduced individually into a cylindrical container (30 cm in height x 10 cm in diameter) and filled with water to a depth of 15 cm thereby presenting them with the challenge of forced swim. The test procedure is based on the assumption that under a stressful situation, each animal expresses escape mobility eventually adopting a characteristic immobile state that is readily identifiable and time recorded. The test was modified such that animals were allowed to swim for 6 min only, the first minute instead of 2 min being considered an acclimatization period while the remaining 5 min were regarded as the test time (Porsolt et al., 1977) and only immobility was recorded.

#### 2.5.5 Analysis of corticosterone serum concentration in parallel with behavioral assessment

The animals were euthanized by cervical dislocation and then decapitated to collect trunk blood in vacutainers without anticoagulant. All the animals in the groups were used to obtain blood samples and brain tissue. Since cervical dislocation was employed, cortical function and biological processes were inhibited as rapidly as possible i.e., 5–10 s (Cartner et al., 2007). Samples were allowed to clot undisturbed for 30–60 min. The clot was removed by centrifugation at 1,500 x g for 15 min at 4°C (5418 R centrifuge, Eppendorf, Hamburg, Germany). Serum samples were assayed immediately or stored at -20°C for subsequent analysis. Corticosterone concentrations were determined using an ELISA kit (Cayman Chemical Company, Ann Arbor, United States, catalogue number 501320), having an assay range of 8.2–5000 pg/ml and a sensitivity (80% B/B_0_) of 30 pg/ml. The corticosterone concentrations in the samples were compared with a standard curve. All samples were thawed on ice and diluted 20-fold in ELISA buffer. A volume of 100 µL ELISA buffer was added to NSB wells (non-specific binding) and 50 µL to B_0_ (maximum binding). A 50 µL corticosterone ELISA standard was then added to each well, followed by 50 µL of corticosterone AChE (Acetylcholinesterase) tracer except for the TA (total activity) and Blk (blank) wells. Finally, a 50 µL corticosterone antiserum was added to each well except for the TA, the NSB and the Blk wells. The plate was covered with plastic film (item No. 400012) and incubated overnight at 4°C. After incubation, each well was emptied and rinsed five times with wash buffer. Next, the wells were filled with Ellman’s Reagent at a volume of 200 µL and 5 µL of tracer to the TA well. The plates were again covered. An orbital shaker equipped with a large flat cover was used to allow the plate to develop in the dark. After 120 min of incubation, the plates were read at 405 nm using a spectrophotometer (T80 + UV/VIS Spectrometer, PG Instruments Limited, Leicestershire, United Kingdom). For standard curve fitting and sample data analysis, a Cayman computer spreadsheet available at (www.caymanchem.com/analysis/elisa) and GraphPad Prism version 8.0 for Windows (GraphPad Software, San Diego, California, United States) were used.

### 2.6 Neurochemical analysis *via* high pressure liquid chromatography (HPLC)

#### 2.6.1 Biological sample preparation

After behavioral experimentation, the mice were euthanized by cervical dislocation and decapitated. The *post mortem* brain regions (frontal cortex, striatum, and hippocampus) were dissected on ice chilled plates where they were accurately weighed and stored at -80°C. The tissues were homogenized *via* a Teflon-glass homogenizer (Ultra-Turax^®^T-50) in 0.2% ice-cold perchloric acid with rpm of 12,000. Eventually all samples were cold centrifuged (4°C) at 12,000 rpm (DLAB Scientific). Later, the supernatant was filtered with 0.45 mm filter (CNW technologies), which was then placed for analysis in an HPLC auto-sampler (Rehman et al., 2020).

#### 2.6.2 Chromatographic conditions

Chromatography was performed (Waters Alliance e2695 separations module with 2998 PDA UV detector, and auto-sampler, United States), using a C18 stainless steel column (250 × 4.6 mm) (Waters X Select^®^ HSS Ireland) with 5 µm particle size. The composition of mobile phase was methanol: HPLC grade water (DAEJUNG; Korea: 8585-2304) in a ratio of 5:95 v/v, plus 20 mM monobasic sodium phosphate (DAEJUNG; Korea: CAS: 7558-80-7) as a buffer. The detection wavelength was 280 nm with isocratic elution. The column temperature was 35°C and flow rate was 0.5 mL/min (Hou et al., 2019; Rehman et al., 2020).

#### 2.6.3 Standard preparation

A standard stock solution of 1.0 mg of dopamine, serotonin, or norepinephrine was prepared by dissolving in 10 mL HPLC grade water. Five different concentrations of 100, 200, 300, 400, 500 ng/mL of stock solution of each neurotransmitter were made by serial dilutions, and then used for the calibration curve. Samples were placed in an HPLC auto-sampler with an injection volume of 20 µL set by software (Empower^TM^). By using linear regression analysis, the calibration curve was plotted using the peak area of dopamine, serotonin, and noradrenaline (y) against the concentration of dopamine, serotonin, and norepinephrine (x) respectively (Rehman et al., 2020).

### 2.7 Statistical analysis

Results were evaluated using GraphPad Prism (version 8). Mean ± SEM was calculated for each group (n = 6/group). Analysis of variance, one-way ANOVA was used followed by *post hoc* Tukey’s test. *p* < 0.05 was considered as a significant value.

## 3 Results

### 3.1 Behavioral analysis

#### 3.1.1 Open field test

**FIGURE 1:**
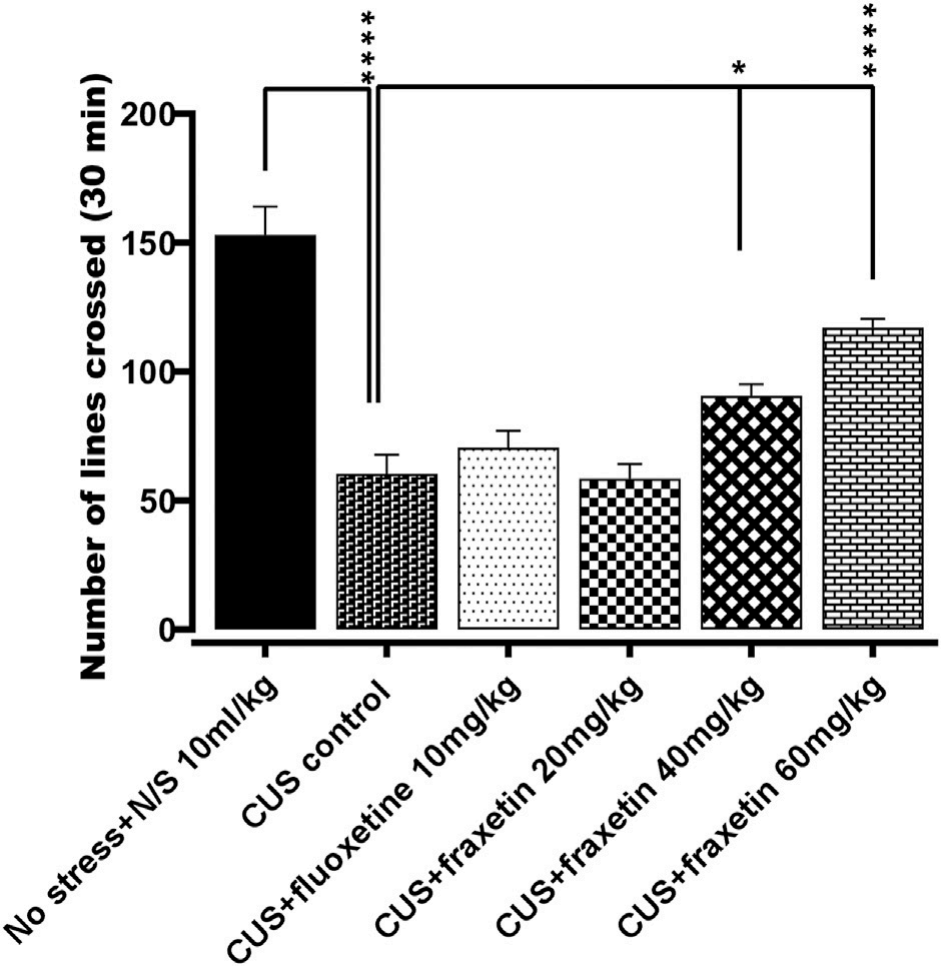
The action of fraxetin (20, 40, and 60 mg/kg, p.o.) or fluoxetine (STD; 10 mg/kg, p.o.) on CUS reduced locomotor activity compared to the vehicle alone treated animal group (saline). Values were expressed as mean ± SEM (*n* = 6/group), *p < 0.05, ****p < 0.0001 vs. CUS. One-way ANOVA was applied followed by *post hoc* Tukey’s test.

There was a substantial decrease in the locomotor activity evoked in the animals exposed to CUS compared to the vehicle alone control treated group (saline) However, this CUS locomotor activity suppression was at least partially restored to levels approaching those of controls by the CUS group co-administered fraxetin 40, and 60 mg/kg F (5, 30) = 29. 43 and *p* < 0.0001. In contrast, there was no significant difference in the degree of locomotion expressed between the animal groups administered fluoxetine (10 mg/kg) as a standard antidepressant or fraxetin (20 mg/kg) (Figure 1).

#### 3.1.2 Elevated plus maze (EPM)

**FIGURE 2:**
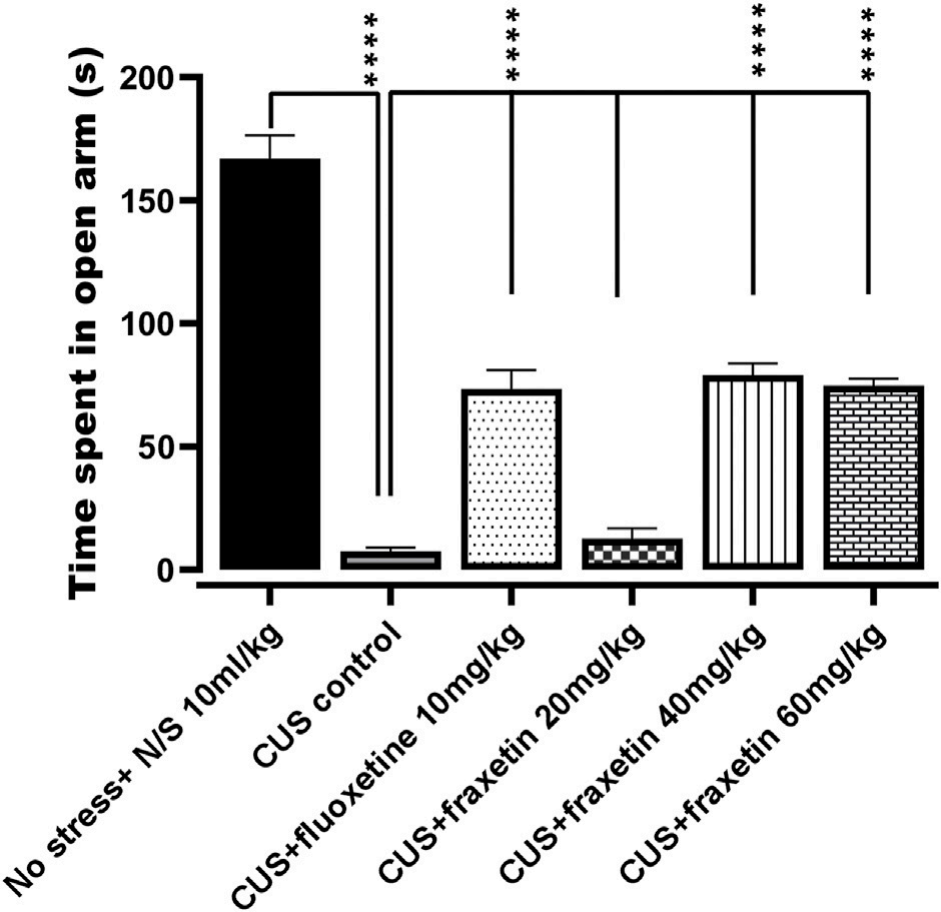
Action of fraxetin (20, 40, and 60 mg/kg, p.o.) or fluoxetine (10 mg/kg, p.o.) on CUS inhibited open arm entry compared to the vehicle alone treated control group (saline) in the EPM test. Values were expressed as mean ± SEM (*n* = 6/group), ****p < 0.0001 vs. CUS. One-way ANOVA was applied followed by post hot Tukey’s test.

In the animal group that underwent CUS, there was an almost total abolition of any time spent in the open EPM arms in comparison with the vehicle-treated controls (saline), and this outcome remained unmodified in the CUS animals co-treated with fraxetin (20 mg/kg). However, combined treatment with fluoxetine (STD) or the two higher doses of fraxetin partially but significantly (Figure 2: F (5, 30) = 98.13 and *p* < 0.0001) restored the CUS inhibition of entry time in the EPM open arms indicating an antianxiety-like effect.

#### 3.1.3 *Y-*maze test (Y-maze)

**FIGURE 3:**
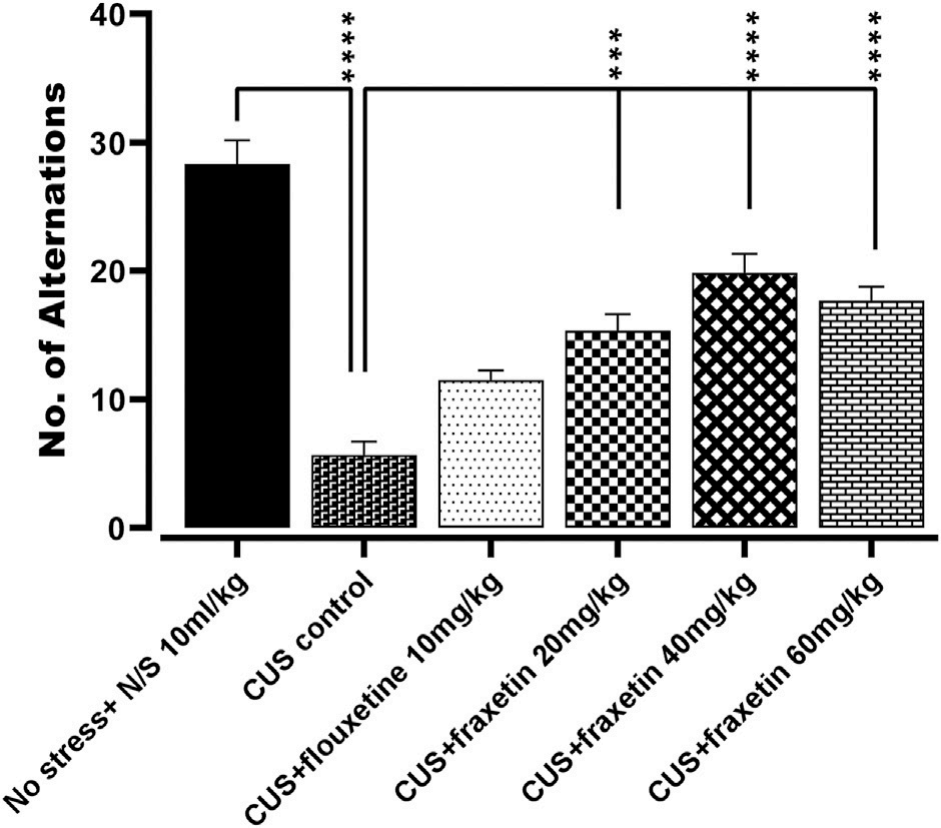
Action of fraxetin (20, 40, and 60 mg/kg, p.o.) or fluoxetine (10 mg/kg, p.o. STD) on CUS evoked reduction in spontaneous number of alternations compared to the vehicle alone treated control group (saline) in the Y-maze. Values were expressed as mean ± SEM (*n* = 6/group), *p < 0.05, ***p < 0.001, ****p < 0.0001 vs. CUS. One-way ANOVA was applied followed by post hot Tukey’s test.

In the animal group that experienced CUS, there was an extensive reduction in the number of spontaneous alternations versus the vehicle alone treated controls (saline) in Y-maze performance indicating an impairment of short-term working memory. Conversely, the CUS repressed incidence of Y-maze arm alternation was significantly redressed by concomitant administration of fluoxetine (STD; 10 mg/kg) and all three doses of fraxetin 10, 20, and 40 mg/kg (Figure 3: F (5, 30) = 35.11 and *p* < 0.0001) reflecting an improvement of spatial memory.

#### 3.1.4 Forced swim test (FST)

**FIGURE 4:**
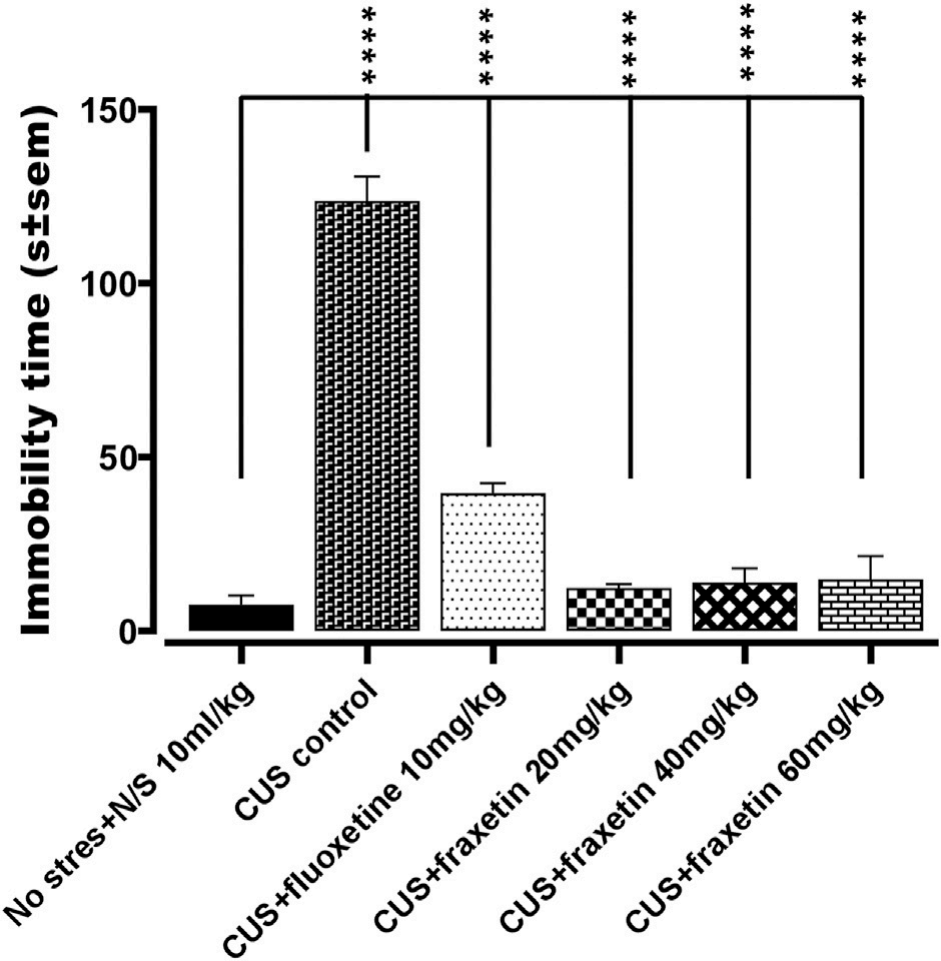
Action of fraxetin (20, 40, and 60 mg/kg, p.o.) or fluoxetine (10 mg/kg, p.o. STD) in the forced swim test (FST): immobility time compared to the vehicle alone treated control group (saline) in the forced swim test. Values were expressed as mean ± SEM (*n* = 6/group), ****p < 0.0001 vs. CUS. One-way ANOVA was applied followed by *post hoc* Tukey’s test.

In the animal group that was subjected to CUS, it was observed that there was an extensive increase in the duration of immobility time (s) versus the vehicle alone treated controls (N/S or vehicle) in the forced swim test, indicating an increase in the behavioral despair. This immobility time duration was markedly reduced by fluoxetine treatment (STD, 10 mg/kg) and totally restored by all three doses of fraxetin to levels that were not significantly different from vehicle-treated controls (saline) Figure 4: F (5, 30) = 94.73 and *p* < 0.0001).

### 3.2 Neurochemical analysis *via* high-pressure liquid chromatography (HPLC)

#### 3.2.1 Action of fraxetin on brain regional serotonin levels

**FIGURE 5:**
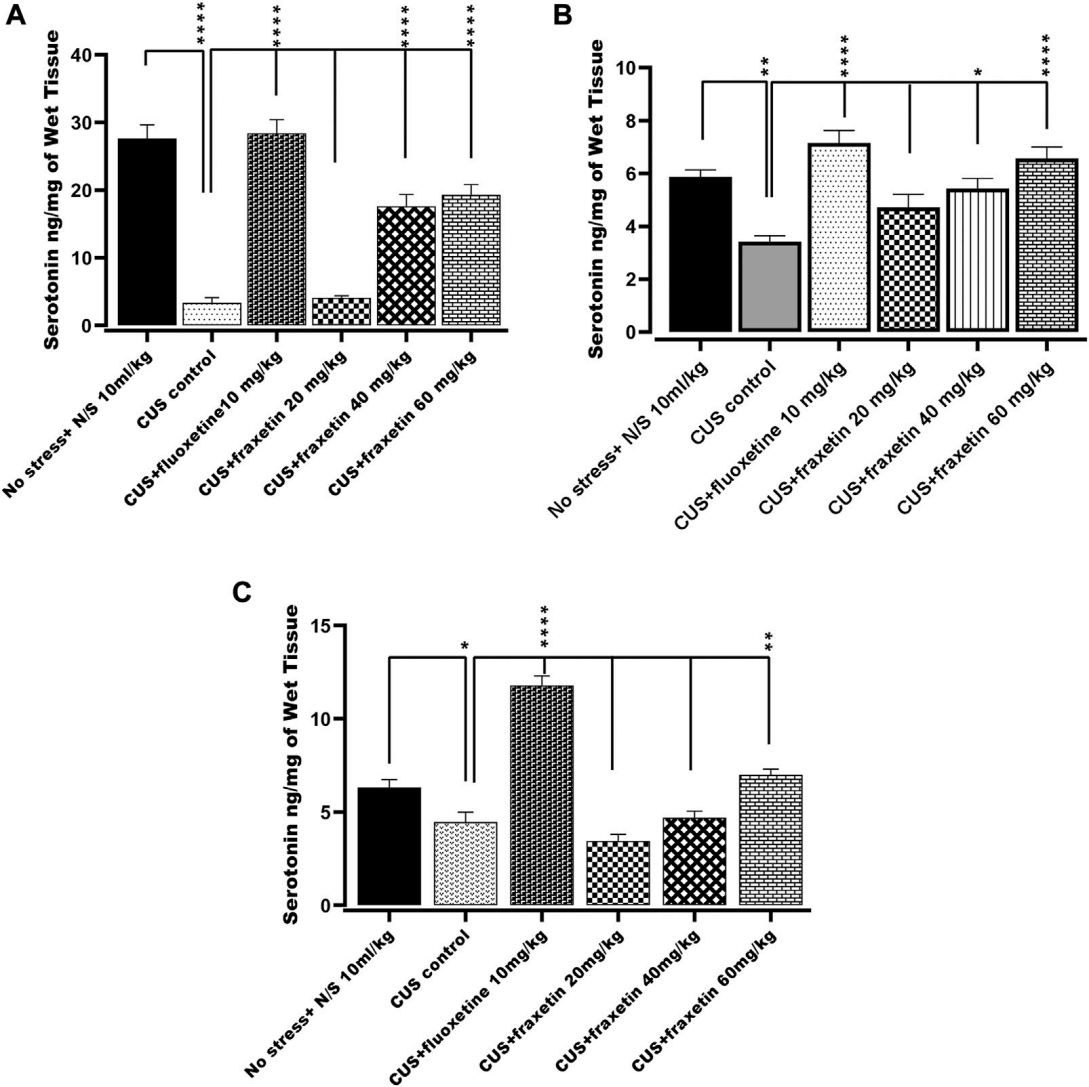
**(A,B)** and **(C)**. Action of fraxetin (20, 40, and 60 mg/kg p.o.) and fluoxetine (10 mg/kg p.o.) on serotonin levels in **(A)** frontal cortex, **(B)** striatum and **(C)** hippocampus in mice subjected to the CUS model. Data were expressed as mean ± SEM (𝑛 = 6/group). *p < 0.05, **p < 0.01, ***p < 0.001 and ****p < 0.0001 compared with the CUS model group. One-way ANOVA was applied with *post hoc* Tukey’s test.

In the CUS exposed group, serotonin levels in the frontal cortex, hippocampus and striatum were decreased significantly as compared with saline-treated non-stressed animals. However, fraxetin (40, and 60 mg/kg) and fluoxetine (10 mg/kg) caused an upsurge in CUS repressed serotonin level in the frontal cortex and striatum F (5, 30) = 50.77 and *p* < 0.0001, while in the hippocampus, fluoxetine (10 mg/kg) and only the highest dose of fraxetin (60 mg/kg) F (5, 30) = 49.92 and *p* < 0.0001 elevated serotonin concentration compared with the CUS group (Figures 5A,B,C).

#### 3.2.2 Action of fraxetin on brain regional dopamine levels

**FIGURE 6:**
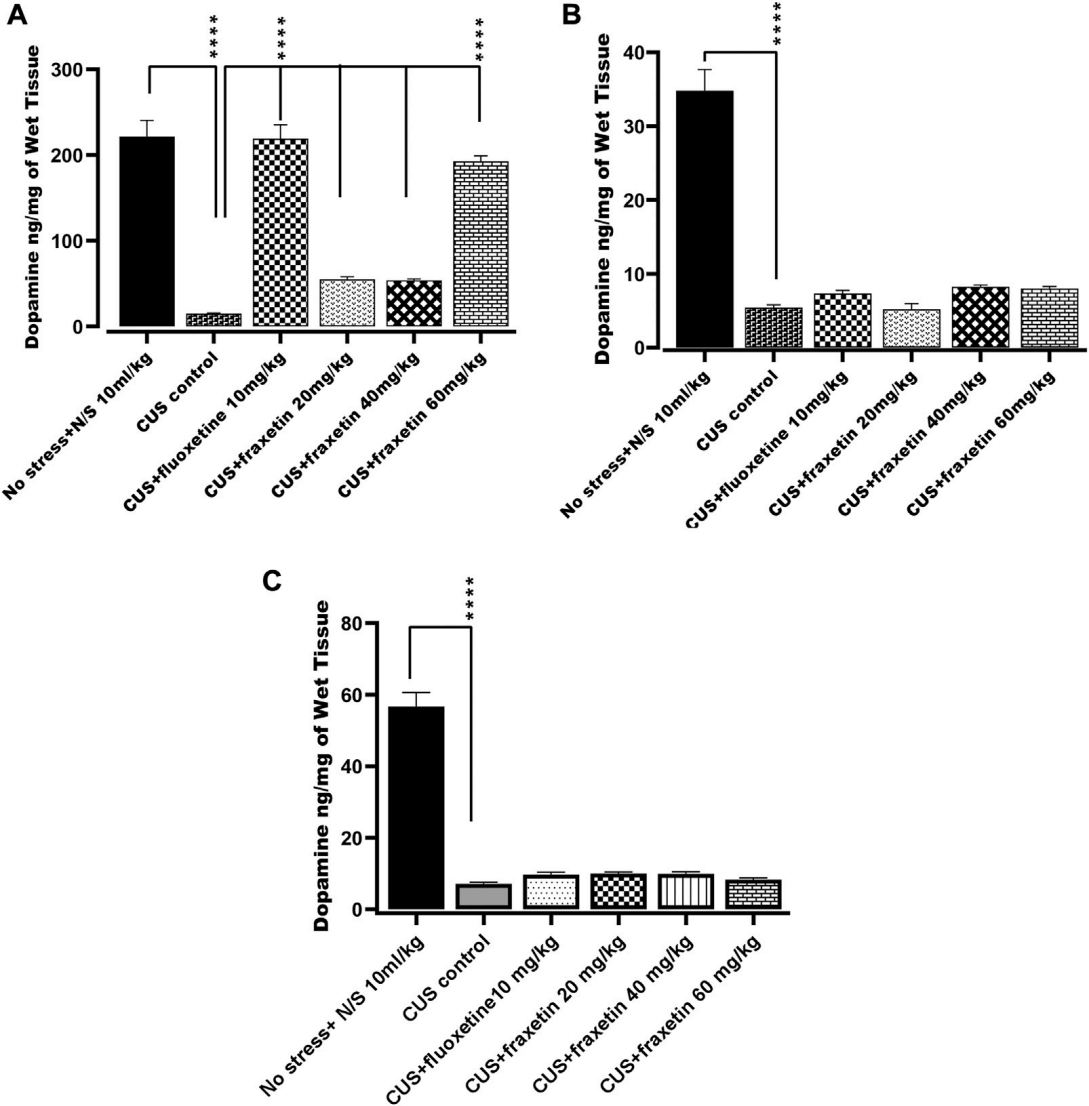
**(A,B)** and **(C)**. Action of fraxetin (20, 40, and 60 mg/kg, p.o.) and fluoxetine 10 mg/kg, p.o.) on dopamine level in **(A)** frontal cortex, **(B)** striatum and **(C)** hippocampus in mice subjected to the CUS model. Data was expressed as mean ± SEM (𝑛 = 6/group). *p < 0.05, **p < 0.01, ***p < 0.001 and ****p < 0.0001 compared with the CUS model group. One-way ANOVA followed by Tukey’s Post hoc multiple comparison test was applied for statistical analysis.

In the group subjected to CUS, dopamine levels in the frontal cortex, hippocampus and striatum were significantly diminished compared with saline-treated non-stressed animals. There was no statistically significant change in the dopamine level in the striatum and hippocampus after treatment with either fraxetin (20, 40, and 60 mg/kg) or fluoxetine (10 mg/kg) in the CUS group, (Figures 6B,C). In contrast, fraxetin (60 mg/kg) and fluoxetine (10 mg/kg) both increased CUS suppressed dopamine concentrations in the frontal cortex to levels which were comparable to the non-stressed animals (Figure 6A: F (5, 30) = 80.58 and *p* < 0.0001).

#### 3.2.3 Action of fraxetin on brain regional norepinephrine levels

**FIGURE 7:**
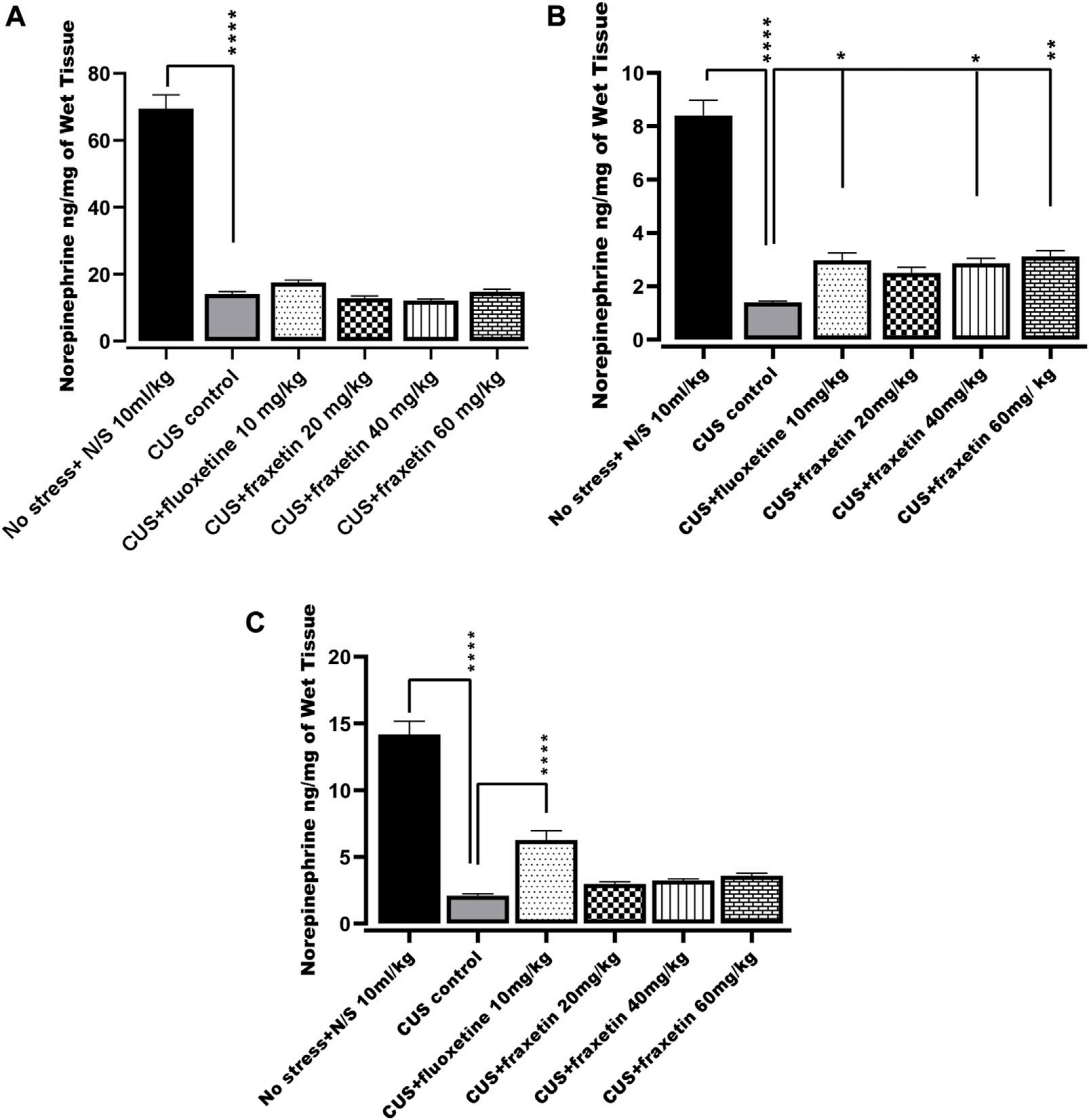
**(A,B)** and **(C)**. Action of fraxetin (20, 40, and 60 mg/kg p.o.) and fluoxetine (10 mg/kg p.o.) on norepinephrine level in **(A)** frontal cortex **(B)** striatum and **(C)** hippocampus in mice subjected to the CUS model. Data were expressed as mean ± SEM (𝑛 = 6/group). *p < 0.05, **p < 0.01, ***p < 0.001 compared with CUS model group. One-way ANOVA was applied followed by Tukey’s multiple comparison test.

In the CUS model group, norepinephrine levels in the frontal cortex, hippocampus, and striatum were markedly decreased compared with the saline-treated non-stressed animals. However, after treatment with fraxetin (40, and 60 mg/kg), norepinephrine levels were elevated in the striatum only (Figure 7B: F (5, 30) = 66.16 and *p* < 0.0001) while fluoxetine 10 mg/kg raised norepinephrine concentrations in both the hippocampus F (5, 30) = 79.26 and *p* < 0.0001 and striatum (Figures 7B,C) compared to the CUS positive control group (Figure 7B).

### 3.3 Corticosterone serum concentration parallel to behavioral assessment

**FIGURE 8:**
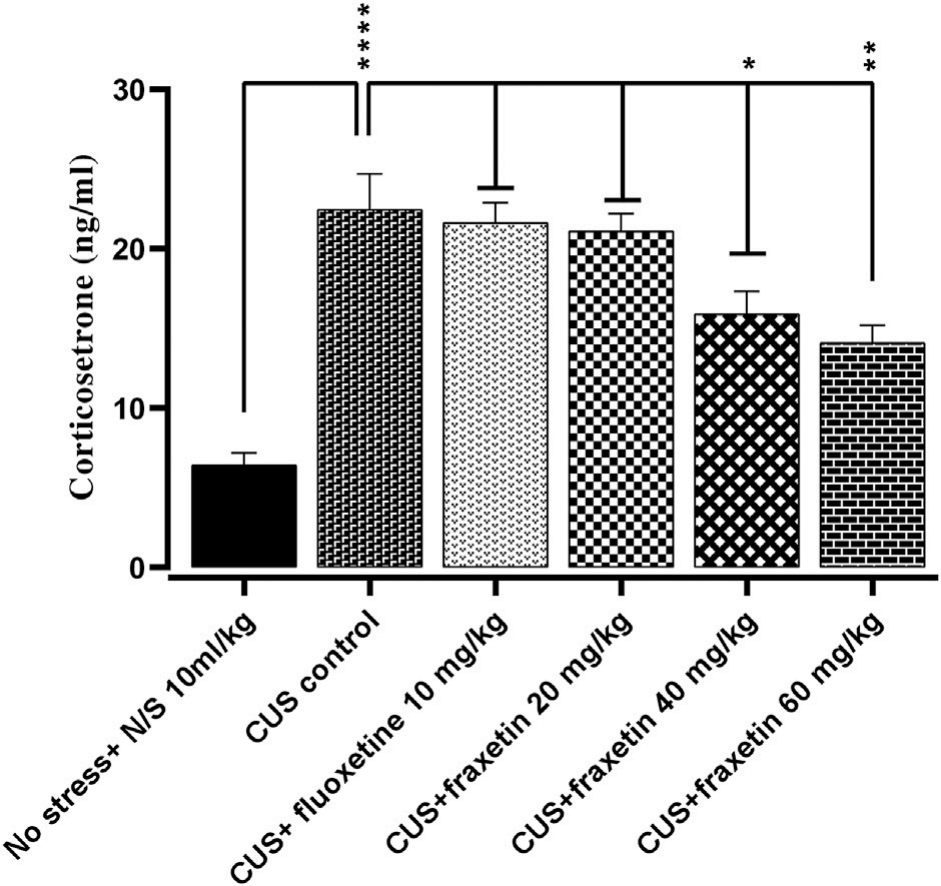
The effects of acute fraxetin (20 mg/kg, 40 mg/kg or 60 mg/kg) on 14 days chronic unpredictable stress on the serum corticosterone levels in mice. The data expressed as the means ± SEMs (N = 6/group). GraphPad Prism was used to analyze the data. *p* < 0.05 was the significance vale.

There was a very highly significantly increased serum concentration of corticosterone in the CUS group compared to the non-stressed control animal group. However, this augmented level of corticosterone was significantly reversed by fraxetin (40 mg/kg and 60 mg/kg) but not by the 20 mg/kg dose (Figure 8: F (5, 30) = 19.51 and *p* < 0.0001).

## 4 Discussion

In the present study, our findings showed that fraxetin significantly attenuated CUS-induced anxiety and depressive-like behaviors in the open field test, elevated plus maze and forced swim test. Moreover, fraxetin also significantly improved spatial learning and working memory in the Y-maze.

Chronic unpredictable stress (CUS) can induce an impairment of cognition and adversely affect emotional aspects such as anxiety, depression, and irritability in addition to diminishing social interaction. Altered serotonergic activity occurring during stress in the central nervous system is a primary and crucial factor contributing to these symptoms. Moreover, a combination of autonomic symptoms including gastrointestinal problems, hyperactivity, restlessness plus paresthesia, and more centrally-mediated symptoms such as binge eating and sleep disorders may also develop. Stressful events are responsible for the release of corticotrophin-releasing factor (CRF) from the hypothalamic paraventricular nucleus (PVN) which in turn releases adrenocorticotropic hormone (ACTH) from the pituitary and ultimately stimulates the adrenal cortex to secrete cortisol in humans and corticosterone in rodents. In addition, stress can lead to perturbation of the blood-brain barrier (BBB) and induction of cellular and neuronal damage, leading to neuronal and cellular inflammation and ultimately death. All of these crucial influential elements can be instrumental in the disruption of cognitive processes (O’connor et al., 2000; Hughes et al., 2016; Sántha et al., 2016). The stress response is a consequence of sympathetic nervous system activation largely dependent on the intensity and duration of stressful events and also through activation of stress pathways in the hypothalamic-pituitary-adrenal axis (Mcewen, 2000; Kim and Diamond, 2002; Sántha et al., 2016; Cameron and Schoenfeld, 2018). Additionally, acute stress may increase memory consolidation by increasing glutamatergic neurotransmission thereby improving spatial memory (Lupien et al., 2002; Roozendaal et al., 2006). In this context, separate groups were run for each behavioral test and acute administration of fluoxetine, fraxetin or vehicle was given after 5 h of the last stressor in order to avoid any additional stress experienced during behavioral testing.

Coumarins and their derivatives have recently attracted much attention regarding their diverse biological and pharmacological properties. On one hand, they offer microbial resistance against pathogens in plants while on the other hand in humans, they are thought to possess aptitude against cancer, pain and inflammation in addition to acting as antimicrobials, anti-Alzheimer, and anti-Parkinsonian agents (Hussain et al., 2019).

Fraxetin, a natural derivative of coumarin, is present in many functional foods and various dietary supplements (Gaur et al., 2017). Furthermore, it displays neuroprotective, biological scavenger/antioxidant, anti-hyperglycemic, antibacterial actions, and also inhibits platelet aggregation along with anti-osteoporotic properties (Sánchez-Reus et al., 2005; Kuo et al., 2006; Wang et al., 2020). Fraxetin readily crosses the blood-brain barrier, maintaining permeability and its antioxidant activity may well have potential in neurodegenerative disorders that stem from oxidative stress (Ng et al., 2000; Cui et al., 2022).

Bearing in mind its neuroprotective and antioxidant profile along with antidepressant properties, the effects of fraxetin were investigated in the CUS plus FST model for depression. Accordingly, CUS exhibited depressive-like behavior in the FST which was disclosed as an increase in the duration of immobility time. Subsequently, acute treatment with fraxetin reversed CUS induced depressive-like behavior as shown in (Figure 4). This finding may suggest that its pharmacological activity might have an overlap with fast-acting antidepressants such as ketamine and brexanolone rather than more conventional monoaminergic-based drugs (Li, 2020). The approval of fast-acting antidepressants i.e., S-ketamine and brexanolone by the FDA in 2019, has provided new insight into treating depression with new targets such as NMDA and GABA_A_ agents (Li, 2020). NMDA receptors (NMDARs) are associated with glutamate-gated ion channels expressed throughout the entire central nervous system providing excitatory synaptic neurotransmission. NMDARs are heteromeric complexes comprising many homologous subunits (Neyton and Paoletti, 2006; Ide and Ikeda, 2020). In this connection, ketamine delivers a rapid and sustained antidepressant effect which is achieved primarily *via* NMDA receptor antagonism and this mechanism may well be shared by fraxetin during its acute antidepressant-like activity in the FST. This assertion is further substantiated by the fact that computationally, fraxetin is an NMDA antagonist at the glutamic acid position 236 and 106 and arginine at position 115 *via* hydrogen bonding, a possible consequence of which is that fraxetin’s fast-acting antidepressant-like property might be ascribed to a selective antagonistic effect at the NMDA receptor (supplementary file attached).

The neuroprotective profile of fraxetin is well documented (Wang et al., 2020), and our findings confirm that acute treatment with all three doses of fraxetin (20, 40, and 60 mg/kg) attenuated CUS-induced memory impairment observed in Y-maze performance (Figure 3). The elevated plus maze (EPM) is a simple method used to assess anxiety, and what is more, it can also be used for the anxiogenic-like effect of pharmacological agents, hormones, and drugs of abuse. In the present study, acute administration of fraxetin (40 and 60 mg/kg) ameliorated CUS-induced anxiety-like behavior in the elevated plus-maze (EPM) implying the possible modulation of GABAergic neurotransmission (Figure 2). In relation to this finding, not only does CUS exacerbate memory loss and the accumulation of hippocampal senescent cells but CUS treatment with a senolytic cocktail containing another phytochemical agent, quercetin, alleviates this cognitive deficit (Lin et al., 2021).

The OFT is a classical approach/avoidance paradigm in which the novel environment concomitantly evokes both anxiety and exploration (Guo et al., 2009). Acute treatment with fraxetin allayed the anxiety-like behavior expressed by the CUS model and there was no stimulant effect of fraxetin on its own in naive mice (unpublished data) (see Figure 1).

Altered monoaminergic neurotransmission involving serotonin, norepinephrine, and dopamine in the initiation and development of various psychiatric disorders, especially depression, has been well-documented (Elhwuegi, 2004; Krishnan and Nestler, 2008), and antidepressants tend to operate either directly or indirectly to restore monoamine levels (Krishnan and Nestler, 2008). The present study was intended to investigate the acute effect of fraxetin in the CUS model of depression for both behavioral as well neurochemical profiling. For this reason, the levels of serotonin, norepinephrine and dopamine were quantified in the frontal cortex, hippocampus, and striatum in animals exposed to CUS compared to those cotreated with fraxetin. These brain regions and neurotransmitters were selected for study because they are known to be involved in the pathogenesis of depression (Drevets et al., 2008).

It was found that in the CUS model, the levels of serotonin, norepinephrine and dopamine in the frontal cortex, hippocampus and striatum were all significantly decreased and these outcomes are consistent with earlier published findings (Yu et al., 2013; Natarajan et al., 2017; Malta et al., 2021). Furthermore, fraxetin and fluoxetine have been reported to activate the Akt/PKB signaling pathway, a serine/threonine-protein kinase that is a critical regulator of cell survival and proliferation including nutrient metabolism, cell growth and apoptosis. Additionally, regulation of this pathway also plays a role as an integrator of serotonin and dopamine neurotransmission with the function of genes linked to disorders of the CNS (Mo et al., 2019; Kitagishi et al., 2012; Beaulieu, 2012).

Acute treatment with fraxetin tended to restore CUS suppressed serotonin levels in the frontal cortex, striatum and hippocampus. Serotonergic pathways throughout the CNS are altered in CUS and there is an imbalance between serotonin levels and receptor densities (Lages et al., 2021). The hippocampus is the main region where alteration of key neurotransmitter pathways and specified protein levels occur in response to CUS and the differential neurotransmitter modification is reflected by variable levels of behavioral expression (Zhang et al., 2021b). Additionally, it was notable that a similar behavioral and neurochemical pattern was seen with standard fluoxetine. These results confirm that acute fraxetin has a significant effect on brain serotonin levels in the frontal cortex, hippocampus and striatum at higher doses and this may explain its antidepressant-like behavior in the FST. Additionally fraxetin also increased norepinephrine concentrations in the striatum, but not in the frontal cortex or hippocampus, while at the same time, only at the highest dose, it did produce an upsurge in dopamine levels in the frontal cortex. Furthermore, sub-acute and chronic studies are warranted to investigate the effects of fraxetin and its interactions with serotoninergic, noradrenergic and dopaminergic receptors to further probe a more precise mechanism.

Together with the neurotransmitter alterations, there was a raised serum concentration of corticosterone in the CUS group compared to non-stressed animals, however, this augmented level of corticosterone was reversed by the higher doses of fraxetin. It is well documented that persistent and prolonged elevation of cortisol in humans can decrease levels of serotonin and a model of depression that has been employed in preclinical studies involves the use of cortisol. Conversely, the finding that fraxetin raised the CUS-suppressed serotonin level may in turn have had an effect resulting in a decline of serum corticosteroid in mice (Figure 8) (Moretti et al., 2012).

## 5 Conclusion

The identification of fast-acting antidepressants with minimal side effects has recently gained special attention in the treatment of depression. A worthwhile approach of this study was an investigation of antidepressant-like properties of fraxetin that might share a possible underlying mechanism with fluoxetine. Thus, we showed that acute fraxetin treatment can attenuate not only depressive- and anxiety-like behavior in the OFT, EPM, Y-maze, and FST paradigms, but also the raised corticosterone along with suppressed serotonin levels subsequent to a CUS protocol. Further, studies are warranted to explore the chronic effect of fraxetin at the molecular level in brain areas associated with depression, anxiety and stress.

## Data Availability

The original contributions presented in the study are included in the article/supplementary material, further inquiries can be directed to the corresponding author.

## References

[B1] Abu-Aisheh M. N. Al-Aboudi A. Mustafa M. S. El-Abadelah M. M. Ali S. Y. Ul-Haq Z. (2019). Coumarin derivatives as acetyl-and butyrylcholinestrase inhibitors: An *in vitro*, molecular docking, and molecular dynamics simulations study. Heliyon 5 (4), e01552. 10.1016/j.heliyon.2019.e01552 31183424 PMC6488543

[B2] An S. H. Choi G.-S. Ahn J.-H. (2020). Biosynthesis of fraxetin from three different substrates using engineered *Escherichia coli* . Appl. Biol. Chem. 63 (1), 55–56. 10.1186/s13765-020-00543-9

[B3] Arif M. Rauf K. Rehman N. U. Tokhi A. Ikram M. Sewell R. D. (2022). 6-Methoxyflavone and donepezil behavioral plus neurochemical correlates in reversing chronic ethanol and withdrawal induced cognitive impairment. Drug Des. Dev. Ther. 16, 1573–1593. 10.2147/DDDT.S360677 PMC9160976 35665194

[B4] Blackburn T. P. (2019). Depressive disorders: Treatment failures and poor prognosis over the last 50 years. Pharmacol. Res. Perspect. 7 (3), e00472. 10.1002/prp2.472 31065377 PMC6498411

[B5] Bondi C. O. Rodriguez G. Gould G. G. Frazer A. Morilak D. A. (2008). Chronic unpredictable stress induces a cognitive deficit and anxiety-like behavior in rats that is prevented by chronic antidepressant drug treatment. Neuropsychopharmacology 33 (2), 320–331. 10.1038/sj.npp.1301410 17406647

[B6] Cameron H. A. Schoenfeld T. J. (2018). Behavioral and structural adaptations to stress. Front. Neuroendocrinol. 49, 106–113. 10.1016/j.yfrne.2018.02.002 29421158 PMC5963997

[B7] Carrasco G. A. Van de Kar L. D. (2003). Neuroendocrine pharmacology of stress. Eur. J. Pharmacol. 463 (1-3), 235–272. 10.1016/s0014-2999(03)01285-8 12600714

[B8] Cartner S. C. Barlow S. C. Ness T. J. (2007). Loss of cortical function in mice after decapitation, cervical dislocation, potassium chloride injection, and CO2 inhalation. Comp. Med. 57 (6), 570–573. 18246869

[B9] Charmandari E. Tsigos C. Chrousos G. (2005). Endocrinology of the stress response. Annu. Rev. Physiol. 67, 259–284. 10.1146/annurev.physiol.67.040403.120816 15709959

[B10] Cui Y. Liu M. Zuo L. Wang H. Liu J. (2022). Fraxetin protects rat brains from the cerebral stroke via promoting angiogenesis and activating PI3K/Akt pathway. Immunopharmacol. Immunotoxicol. 44 (3), 400–409. 10.1080/08923973.2022.2052893 35285387

[B11] Daliev B. B. Bychkov E. R. Myznikov L. V. Lebedev A. A. Shabanov P. D. (2021). Anticompulsive effects of novel derivatives of coumarin in rats. Rev. Clin. Pharmacol. Drug Ther. 19 (3), 339–344. 10.17816/rcf193339-344

[B12] Drevets W. C. Price J. L. Furey M. L. (2008). Brain structural and functional abnormalities in mood disorders: Implications for neurocircuitry models of depression. Brain Struct. Funct. 213 (1-2), 93–118. 10.1007/s00429-008-0189-x 18704495 PMC2522333

[B13] Duman R. S. Aghajanian G. K. (2012). Synaptic dysfunction in depression: Potential therapeutic targets. science 338 (6103), 68–72. 10.1126/science.1222939 23042884 PMC4424898

[B14] Elhwuegi A. S. (2004). Central monoamines and their role in major depression. Prog. Neuro-Psychopharmacology Biol. Psychiatry 28 (3), 435–451. 10.1016/j.pnpbp.2003.11.018 15093950

[B15] Gaur P. Singh D. K. Luqman S. Shanker K. (2017). Validated method for quality assessment of henna (Lawsonia inermis L.) leaves after postharvest blanching and its cosmetic application. Industrial Crops Prod. 95, 33–42. 10.1016/j.indcrop.2016.10.010

[B16] Gold P. W. (2015). The organization of the stress system and its dysregulation in depressive illness. Mol. psychiatry 20 (1), 32–47. 10.1038/mp.2014.163 25486982

[B17] Golub H. M. Zhou Q. G. Zucker H. McMullen M. R. Kokiko-Cochran O. N. Ro E. J. (2015). Chronic alcohol exposure is associated with decreased neurogenesis, aberrant integration of newborn neurons, and cognitive dysfunction in female mice. Alcohol. Clin. Exp. Res. 39 (10), 1967–1977. 10.1111/acer.12843 26365148 PMC4592440

[B18] Guo J.-Y. Li C.-Y. Ruan Y.-P. Sun M. Qi X.-L. Zhao B.-S. (2009). Chronic treatment with celecoxib reverses chronic unpredictable stress-induced depressive-like behavior via reducing cyclooxygenase-2 expression in rat brain. Eur. J. Pharmacol. 612 (1-3), 54–60. 10.1016/j.ejphar.2009.03.076 19356723

[B19] Habib K. E. Gold P. W. Chrousos G. P. (2001). Neuroendocrinology of stress. Endocrinol. Metabolism Clin. 30 (3), 695–728. 10.1016/s0889-8529(05)70208-5 11571937

[B20] Handley S. L. Mithani S. (1984). Effects of alpha-adrenoceptor agonists and antagonists in a maze-exploration model of ‘fear’-motivated behaviour. Naunyn-Schmiedeberg's archives Pharmacol. 327 (1), 1–5. 10.1007/BF00504983 6149466

[B21] Hou X. Huang W. Tong Y. Tian M. (2019). Hollow dummy template imprinted boronate-modified polymers for extraction of norepinephrine, epinephrine and dopamine prior to quantitation by HPLC. Microchim. Acta 186 (11), 686–689. 10.1007/s00604-019-3801-2 31595360

[B22] Hughes M. M. Connor T. J. Harkin A. (2016). Stress-related immune markers in depression: Implications for treatment. Int. J. Neuropsychopharmacol. 19 (6), pyw001. 10.1093/ijnp/pyw001 26775294 PMC4926799

[B23] Hussain M. I. Syed Q. A. Khattak M. N. K. Hafez B. Reigosa M. J. El-Keblawy A. (2019). Natural product coumarins: Biological and pharmacological perspectives. Biologia 2019, 1–26. 10.2478/s11756-019-00242-x

[B24] Ide S. Ikeda K. (2020). “Distinct roles of NMDA receptor GluN2 subunits in the effects of ketamine and its enantiomers,” in Ketamine (Singapore: Springer), 157–173.

[B25] Kılıç C. S. (2022). “Herbal coumarins in healthcare,” in Herbal biomolecules in healthcare applications (Netherlands: Elsevier), 363–380.

[B26] Kim J. J. Diamond D. M. (2002). The stressed hippocampus, synaptic plasticity and lost memories. Nat. Rev. Neurosci. 3 (6), 453–462. 10.1038/nrn849 12042880

[B27] Koyiparambath V. P. Prayaga Rajappan K. Rangarajan T. Al-Sehemi A. G. Pannipara M. Bhaskar V. (2021). Deciphering the detailed structure–activity relationship of coumarins as monoamine oxidase enzyme inhibitors—an updated review. Chem. Biol. Drug Des. 98 (4), 655–673. 10.1111/cbdd.13919 34233082

[B28] Krishnan V. Nestler E. J. (2008). The molecular neurobiology of depression. Nature 455 (7215), 894–902. 10.1038/nature07455 18923511 PMC2721780

[B29] Kryst J. Majcher-Maślanka I. Chocyk A. (2022). Effects of chronic fluoxetine treatment on anxiety-and depressive-like behaviors in adolescent rodents–systematic review and meta-analysis. Pharmacol. Rep. 74 (5), 920–946. 10.1007/s43440-022-00420-w 36151445 PMC9584991

[B30] Kuo P.-L. Huang Y.-T. Chang C.-H. Chang J.-K. (2006). Fraxetin inhibits the induction of anti-Fas IgM, tumor necrosis factor-alpha and interleukin-1beta-mediated apoptosis by Fas pathway inhibition in human osteoblastic cell line MG-63. Int. Immunopharmacol. 6 (7), 1167–1175. 10.1016/j.intimp.2006.02.010 16714221

[B31] Lages Y. Rossi A. Krahe T. Landeira-Fernandez J. (2021). Effect of chronic unpredictable mild stress on the expression profile of serotonin receptors in rats and mice: A meta-analysis. Neurosci. Biobehav. Rev. 124, 78–88. 10.1016/j.neubiorev.2021.01.020 33524415

[B32] Lee A. L. Ogle W. O. Sapolsky R. M. (2002). Stress and depression: Possible links to neuron death in the hippocampus. Bipolar Disord. 4 (2), 117–128. 10.1034/j.1399-5618.2002.01144.x 12071509

[B33] Li J.-M. Zhang X. Wang X. Xie Y.-C. Kong L.-D. (2011). Protective effects of cortex fraxini coumarines against oxonate-induced hyperuricemia and renal dysfunction in mice. Eur. J. Pharmacol. 666 (1-3), 196–204. 10.1016/j.ejphar.2011.05.021 21620826

[B34] Li Y.-F. (2020). A hypothesis of monoamine (5-ht)–glutamate/GABA long neural circuit: Aiming for fast-onset antidepressant discovery. Pharmacol. Ther. 208, 107494. 10.1016/j.pharmthera.2020.107494 31991195

[B35] Lin Y.-F. Wang L.-Y. Chen C.-S. Li C.-C. Hsiao Y.-H. (2021). Cellular senescence as a driver of cognitive decline triggered by chronic unpredictable stress. Neurobiol. stress 15, 100341. 10.1016/j.ynstr.2021.100341 34095365 PMC8163993

[B36] Lupien S. J. Wilkinson C. W. Brière S. Ménard C. Kin N. N. Y. Nair N. (2002). The modulatory effects of corticosteroids on cognition: Studies in young human populations. Psychoneuroendocrinology 27 (3), 401–416. 10.1016/s0306-4530(01)00061-0 11818174

[B37] Malta M. B. Martins J. Novaes L. S. dos Santos N. B. Sita L. V. Camarini R. (2021). Norepinephrine and glucocorticoids modulate chronic unpredictable stress-induced increase in the type 2 CRF and glucocorticoid receptors in brain structures related to the HPA axis activation. bioRxiv. 10.1007/s12035-021-02470-2 34213722

[B38] McEwen B. S. (2000). The neurobiology of stress: From serendipity to clinical relevance. Brain Res. 886 (1-2), 172–189. 10.1016/s0006-8993(00)02950-4 11119695

[B39] Moghaddam B. Javitt D. (2012). From revolution to evolution: The glutamate hypothesis of schizophrenia and its implication for treatment. Neuropsychopharmacology 37 (1), 4–15. 10.1038/npp.2011.181 21956446 PMC3238069

[B40] Moretti M. Colla A. de Oliveira Balen G. dos Santos D. B. Budni J. de Freitas A. E. (2012). Ascorbic acid treatment, similarly to fluoxetine, reverses depressive-like behavior and brain oxidative damage induced by chronic unpredictable stress. J. psychiatric Res. 46 (3), 331–340. 10.1016/j.jpsychires.2011.11.009 22154133

[B41] Moretti M. de Freitas A. E. Budni J. Fernandes S. C. P. de Oliveira Balen G. Rodrigues A. L. S. (2011). Involvement of nitric oxide–cGMP pathway in the antidepressant-like effect of ascorbic acid in the tail suspension test. Behav. Brain Res. 225 (1), 328–333. 10.1016/j.bbr.2011.07.024 21802450

[B42] Murali R. Srinivasan S. Ashokkumar N. (2013). Antihyperglycemic effect of fraxetin on hepatic key enzymes of carbohydrate metabolism in streptozotocin-induced diabetic rats. Biochimie 95 (10), 1848–1854. 10.1016/j.biochi.2013.06.013 23806420

[B43] Natarajan R. Forrester L. Chiaia N. L. Yamamoto B. K. (2017). Chronic-stress-induced behavioral changes associated with subregion-selective serotonin cell death in the dorsal raphe. J. Neurosci. 37 (26), 6214–6223. 10.1523/JNEUROSCI.3781-16.2017 28546314 PMC5490060

[B44] Nestler E. J. Barrot M. DiLeone R. J. Eisch A. J. Gold S. J. Monteggia L. M. (2002). Neurobiology of depression. Neuron 34 (1), 13–25. 10.1016/s0896-6273(02)00653-0 11931738

[B45] Neyton J. Paoletti P. (2006). Relating NMDA receptor function to receptor subunit composition: Limitations of the pharmacological approach. J. Neurosci. 26 (5), 1331–1333. 10.1523/JNEUROSCI.5242-05.2006 16452656 PMC6675501

[B46] Ng T. Liu F. Wang Z. (2000). Antioxidative activity of natural products from plants. Life Sci. 66 (8), 709–723. 10.1016/s0024-3205(99)00642-6 10680579

[B47] O'connor T. O'halloran D. Shanahan F. (2000). The stress response and the hypothalamic-pituitary-adrenal axis: From molecule to melancholia. Qjm 93 (6), 323–333. 10.1093/qjmed/93.6.323 10873181

[B48] Porsolt R. Bertin A. Jalfre M. (1977). Behavioral despair in mice: A primary screening test for antidepressants. Archives Int. de pharmacodynamie de Ther. 229 (2), 327–336. 596982

[B49] Pruccoli L. (2019). Neuroprotective effects of coumarins in neurodegenerative disease models. Science 31, 8975. 10.48676/unibo/amsdottorato/8975

[B50] Qin Z. Zhang B. Yang J. Li S. Xu J. Yao Z. (2019). The efflux mechanism of fraxetin-O-glucuronides in ugt1a9-transfected HeLa cells: Identification of multidrug resistance-associated proteins 3 and 4 (MRP3/4) as the important contributors. Front. Pharmacol. 10, 496. 10.3389/fphar.2019.00496 31133859 PMC6515931

[B51] Rajkowska G. Miguel-Hidalgo J. J. Wei J. Dilley G. Pittman S. D. Meltzer H. Y. (1999). Morphometric evidence for neuronal and glial prefrontal cell pathology in major depression. Biol. psychiatry 45 (9), 1085–1098. 10.1016/s0006-3223(99)00041-4 10331101

[B52] Rehman N. U. Abbas M. Al-Rashida M. Tokhi A. Arshid M. A. Khan M. S. (2020). Effect of 4-fluoro-N-(4-Sulfamoylbenzyl) benzene sulfonamide on acquisition and expression of nicotine-induced behavioral sensitization and striatal adenosine levels. Drug Des. Dev. Ther. 14, 3777–3786. 10.2147/DDDT.S270025 PMC7505708 32982182

[B53] Roozendaal B. Okuda S. Van der Zee E. A. McGaugh J. L. (2006). Glucocorticoid enhancement of memory requires arousal-induced noradrenergic activation in the basolateral amygdala. Proc. Natl. Acad. Sci. 103 (17), 6741–6746. 10.1073/pnas.0601874103 16611726 PMC1458951

[B54] Sacher J. Neumann J. Fünfstück T. Soliman A. Villringer A. Schroeter M. L. (2012). Mapping the depressed brain: A meta-analysis of structural and functional alterations in major depressive disorder. J. Affect. Disord. 140 (2), 142–148. 10.1016/j.jad.2011.08.001 21890211

[B55] Sánchez-Reus M. I. Peinado I. I. Molina-Jiménez M. F. Benedí J. (2005). Fraxetin prevents rotenone-induced apoptosis by induction of endogenous glutathione in human neuroblastoma cells. Neurosci. Res. 53 (1), 48–56. 10.1016/j.neures.2005.05.009 15996779

[B56] Sántha P. Veszelka S. Hoyk Z. Mészáros M. Walter F. R. Tóth A. E. (2016). Restraint stress-induced morphological changes at the blood-brain barrier in adult rats. Front. Mol. Neurosci. 8, 88. 10.3389/fnmol.2015.00088 26834555 PMC4712270

[B57] Sapolsky R. M. Romero L. M. Munck A. U. (2000). How do glucocorticoids influence stress responses? Integrating permissive, suppressive, stimulatory, and preparative actions. Endocr. Rev. 21 (1), 55–89. 10.1210/edrv.21.1.0389 10696570

[B58] Sharifi-Rad J. Cruz-Martins N. López-Jornet P. Lopez E. P.-F. Harun N. Yeskaliyeva B. (2021). Natural coumarins: Exploring the pharmacological complexity and underlying molecular mechanisms. Oxidative Med. Cell. Longev. 2021, 6492346. 10.1155/2021/6492346 PMC8440074 34531939

[B59] Skalicka-Woźniak K. Orhan I. E. Cordell G. A. Nabavi S. M. Budzyńska B. (2016). Implication of coumarins towards central nervous system disorders. Pharmacol. Res. 103, 188–203. 10.1016/j.phrs.2015.11.023 26657416

[B60] Stetler C. Miller G. E. (2011). Depression and hypothalamic-pituitary-adrenal activation: A quantitative summary of four decades of research. Psychosom. Med. 73 (2), 114–126. 10.1097/PSY.0b013e31820ad12b 21257974

[B61] Wang Q. Zhuang D. Feng W. Ma B. Qin L. Jin L. (2020). Fraxetin inhibits interleukin-1β-induced apoptosis, inflammation, and matrix degradation in chondrocytes and protects rat cartilage *in vivo* . Saudi Pharm. J. 28 (12), 1499–1506. 10.1016/j.jsps.2020.09.016 33424243 PMC7783108

[B62] Yu Y. Wang R. Chen C. Du X. Ruan L. Sun J. (2013). Antidepressant-like effect of trans-resveratrol in chronic stress model: Behavioral and neurochemical evidences. J. psychiatric Res. 47 (3), 315–322. 10.1016/j.jpsychires.2012.10.018 23174668

[B63] Zhang T. Zhang X. Liu N. Ren S. Xia C. Yang X. (2021b). Comparative proteomic characterization of ventral Hippocampus in susceptible and resilient rats subjected to chronic unpredictable stress. Front. Neurosci. 15, 675430. 10.3389/fnins.2021.675430 34220431 PMC8249003

[B64] Zhang T. Zhou B. Sun J. Song J. Nie L. Zhu K. (2021a). Fraxetin suppresses reactive oxygen species-dependent autophagy by the PI3K/Akt pathway to inhibit isoflurane-induced neurotoxicity in hippocampal neuronal cells. J. Appl. Toxicol. 42, 617–628. 10.1002/jat.4243 34553399

